# Broncho Vaxom (OM-85) modulates rhinovirus docking proteins on human airway epithelial cells via Erk1/2 mitogen activated protein kinase and cAMP

**DOI:** 10.1371/journal.pone.0188010

**Published:** 2017-11-28

**Authors:** Michael Roth, Christian Pasquali, Daiana Stolz, Michael Tamm

**Affiliations:** 1 Pulmonary Cell Research, DBM University Basel and Pneumology Clinic, University Hospital Basel, Basel, Switzerland; 2 OM Pharma SA, Geneva, Switzerland; Centre National de la Recherche Scientifique, FRANCE

## Abstract

**Background:**

Bronchial epithelial cells (BEC) are primary target for Rhinovirus infection through attaching to cell membrane proteins. OM-85, a bacterial extract, improves recovery of asthma and COPD patients after viral infections, but only part of the mechanism was addressed, by focusing on defined immune cells.

**Objective:**

We therefore determined the effect of OM-85 on isolated primary human BEC of controls (n = 8), asthma patients (n = 10) and COPD patients (n = 9).

**Methods:**

BEC were treated with OM-85 alone (24 hours) or infected with Rhinovirus. BEC survival was monitored by manual cell counting and Rhinovirus replication by lytic activity. Immuno-blotting and ELISA were used to determine the expression of Rhinovirus interacting proteins: intracellular adhesion molecule (ICAM), major histocompatibility complex class II (MHC-2), complement component C1q receptor (C1q-R), inducible T-Cell co-stimulator (ICOS), its ligand ICOSL, and myeloid differentiation primary response gene 88 (Myd88); as well as for signal transducers Erk1/2, p38, JNK mitogen activated protein kinases MAPK), and cAMP.

**Results:**

OM-85 significantly reduced Rhinovirus-induced BEC death and virus replication. OM-85 significantly increased the expression of virus interacting proteins C1q-R and β-defensin in all 3 probes and groups, which was prevented by either Erk1/2 MAPK or cAMP inhibition. In addition, OM-85 significantly reduced Rhinovirus induced expression of ICAM1 involving p38 MAPK. In BEC OM-85 had no significant effect on the expression of ICOS, ICOSL and MHC-2 membrane proteins nor on the adaptor protein MyD88.

**Conclusion:**

The OM-85-induced increased of C1q-R and β-defensin, both important for antigen presentation and phagocytosis, supports its activity in host cell’s defence against Rhinovirus infection.

## Introduction

Bacterial and viral infections are the major cause of acute exacerbations in asthma and COPD, which leads to worsening of the disease. The most frequent viral infections of the upper airways are *Rhinovirus* (RV), *Influenza virus* and *Respiratory syncytial virus*, which mainly affect young children and elderlies [1]. Preventive measures such as immunisations helped to reduce viral infections but are not commonly practiced. Bacterial extracts such as Broncho Vaxom (OM-85) reduced the duration of viral infections and improved the recovery phase in COPD patients, childhood asthma and elderly patients [2–5]. However, despite increasing data based on experimental models [6–13], the mechanism by which OM-85 reduces viral infections and improves recovery is not fully understood. It can be hypothesised that OM-85 either prepares the immune competent cells to combat with viral infections or it increases the primary host defence of bronchial epithelial cells (BEC) [14].

OM-85 is a mixture of protein extracts obtained from 8 bacteria strains originating from 5 genera and is administrated orally. In COPD patients OM-85 reduced rate of exacerbation and lower respiratory tract infections [5]. The evidence for potential improvement of anti-viral response by OM-85 has been reported in animal models and in humans [15, 16]. The mechanism how orally administered OM-85 improves the response of the lung may be explained by the observation that oral vaccines can pass un-modified through M-cells and Peyers patches in the gut and enter the lymphatic system or activate immune competent cells [17]. This mechanism is supported by studies showing that nano-particles and proteins are transported through the epithelium by specialized epithelial cells without being modified and then act on sup-epithelial cells [18, 19].

Regarding its immune modulatory action, orally applied OM-85 significantly reduced the infection rate of H1N1-influenza virus and of salmonella by unspecific activation of immune reactive cells in animal models [6], and involved the induction of FoxP3^+^T-cell formation and activity [7]. The immune modulatory effect of OM-85 may also be regulated by the reduction Th-2 cell cytokines in favour of Th-1 cytokines, which was shown in rats [8]. In a mouse model OM-85 reduced the expression of the high affinity IgE receptor which will reduce the response to allergens [9, 10]. The latter effect was confirmed in patients with allergic diseases, where OM-85 significantly reduced the level of circulating IgE in patients with allergies [20, 21].

*In vitro*, OM-85 has been shown to activate several intercellular signalling pathways including Erk1/2, mitogen activated protein kinase (MAPK) and NFκB [22]. Thereby, OM-85 increased the expression of β-defensin, IgG and IgA [23, 24]. In regard to host defence, β-defensin plays a major role to recognise micro-organisms and marked them for phagocytosis [24]. In addition, β-defensin affects the expression of the intracellular adhesion molecule (ICAM) which acts as a docking protein for at least RV and can be induced by OM-85 in phagocytic cells [25]. In mice, OM-85 reduced RV infection through this mechanism [26]. This observation may be important for the preventive use of OM-85 in asthma, where ICAM-1 mediated RV induced inflammation [27]. Therefore, we postulated that OM-85 may help to improve host defence by up-regulating β-defensin expression on epithelial cells, which are the primary and major target of viral infections [28].

OM-85 has also been reported to affect other virus binding cell surface proteins on epithelial cells such as the inducible T-cell co-stimulator (ICOS) and its ligand ICOSL [29]. A similar mechanism was described in human thymus epithelial cells and in alveolar epithelial cells where ICOSL was essential for antigen presentation to lymphocytes [30]. Both studies linked the expression of ICOSL to ICAM-1 and major histocompatibility protein-1 (MHC1), which mediates antigen presentation. The host defence system is also activated through the cC1qR (calreticulin, CD93), which binds bacterial, viral and parasitic proteins to induce phagocytosis [31].

In conclusion, OM-85 may improve the host defence system through several cell surface proteins which recognise and help to remove micro-organisms. Therefore, we assess the effect of OM-85 on the expression of the above described cell surface proteins by human primary BEC from patients with asthma or COPD and controls. In addition, we determined the intercellular signalling pathways regulating the expression of these host defence proteins.

## Materials and methods

### Patients

Healthy (n = 8), asthma (n = 10), and COPD (n = 9). Healthy control patients were undergoing diagnostic bronchial biopsy for other reasons than asthma or COPD. Asthma patients have been classified as Mild—sever according to GINA guidelines. COPD patients were classified according to GOLD guidelines (Table 1).

**Table 1:** Patient information.

diagnosis	age	FEV1	gender
**Control**	29	ND	female
	55	ND	female
	53	ND	male
	39	ND	female
	64	ND	male
	63	ND	female
	80	ND	male
	54	ND	female
**Mean (S.E.M.)**	58.29 (3.06)		
**Asthma**	59	49	male
	59	70	female
	49	65	male
	42	87	male
	85	94	female
	51	105	male
	29	78	female
	65	82	female
	49	112	male
	17	82	female
**Mean (S.E.M.)**	50.4 (4.47)	82.3 (5.58)	
**COPD**	73	70	male
	74	65	male
	56	51	female
	70	49	male
	75	32	male
	48	58	female
	65	51	male
	65	66	male
	67	24	male
**Mean (S.E.M.)**	64.0 (2.13)	51.78 (3.84)	

Patient information: NA: not available; ND: not determined

The ethical approval to obtain the required tissue samples exists as a general permission to use not needed biopsy material for scientific studies after each patient gave written informed consent (EKBB 05/06).

### OM-85

A standard solution of OM-85 was provided by OM Pharma SA, 1217 Meyrin 1, Switzerland

### Bronchial epithelial cell (BEC) isolation and characterisation

**Fig 1:**
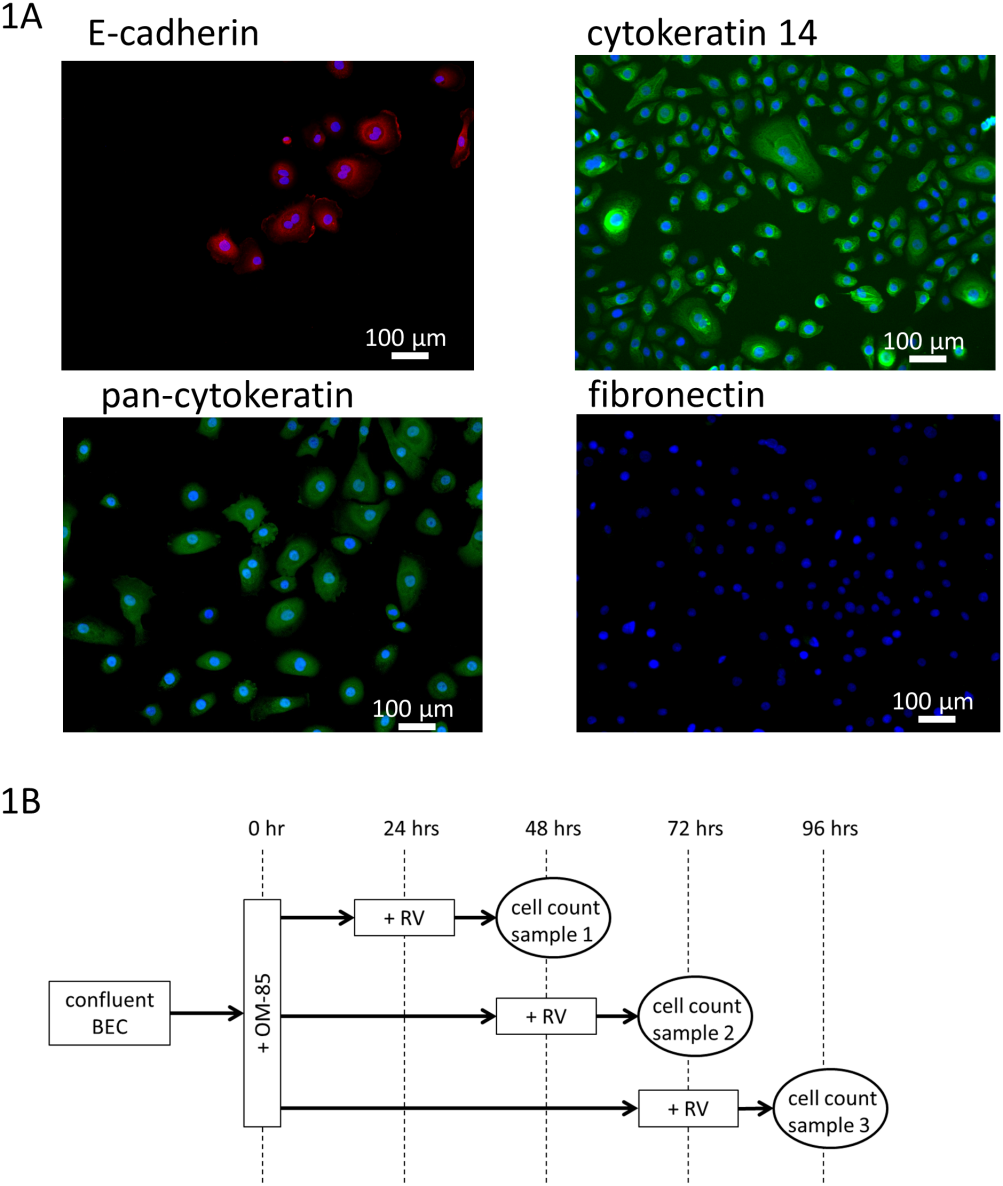
(A) BEC characterisation by IF-staining for: E-cadherin, cytokeratin-14, pan-cytokeratin and negative staining for fibronectin. Images were obtained by EVOS microscope (ThermoFisher Scientific, Switzerland). (B) Treatment schemata for BEC treatment with OM-85 and RV infection.

This has been published earlier [32]. In brief, small pieces (2 x 2 x 2 mm) of bronchial tissues were placed into cell culture vessels, which were pre-wetted with BEC specific medium Cnt-PR-A (CellnTech, Bern, Switzerland). The medium was replaced every second day and BEC were passaged by trypsin/EDTA treatment (5 min., 37°C), before being re-suspended in 3 ml of Cnt-PR-A containing 25% fetal calf serum for better adherence. The adherence medium was replaced after 18 hrs by Cnt-PR-A. BEC were characterised by positive staining of E-Cadherin (Abcam 15148, Abcam, Cambridge, U.K.), pan-cytokeratin (sc-8018, Santa Cruz Biotechnology, Santa Cruz, CA, U.S.A.), cytokeratin-14 (Abcam 9220) and negative staining for fibronectin (Abcam 23751) as shown in Fig 1A.

### Rhinovirus (RV) infection and determination of infection rate

The RV strain used for the experiments has been described earlier [33] and was identified as RV-16. For the experiments described below, we have used the same stock described earlier [33].

BEC were used in passage 1 or 2 and were infected with 1x multiplicity of infection (MOI) of RV for up to 3 days. The infection rate was determined by immunofluorescence staining using an anti-RV16 antibody (cat# 18758, QED-Bioscience Inc. San Diego, USA). Cells were seeded into 8-well chamber slides (Thermofisher Scientific, Switzerland) and treated with RV and other chemical compounds or OM-85, as indicated in the treatment schemata provided in Fig 1B.

After treatment, the cells were fixed after washing with PBS in 4% formalin (in PBS) for 5 minutes. The fixed cells were washed 2x with PBS and permeabilised for 15 minutes with 0.01% TWEEN-100 in PBS. Unspecific binding was blocked in 2% bovine serum albumin (30 minutes in PBS) before being incubated overnight (4°C) with the anti-RV16 antibody (1:100 dilution). Following 3x washes with PBS, cells were incubated with an anti-mouse FITC labelled antibody (Abcam, Switzerland) for 1 hour at room temperature. After 3x washes with PBS, the number of RV positive cells was counted by immunofluorescence microscopy (EVOS FLoid cell imaging station, Thermofisher Scientific) and nuclei were stained for cell counting using the distributor’s live cell reagent (Thermofisher Scientific).

### Immuno-blotting

Protein analysis has been described earlier [33]. Following protein separation through a gradient polyacrylamide gel (4–12%) and electro-blotting onto a PVDF membrane, proteins were identified and their expression rate determined by Western-blotting. The following proteins were detected: β-defensin, C1qR, ICAM1, CREB, and phos-CREB, Erk1/2, phos-Erk1/2, ICOS, ICOSL, JNK, phos-JNK, MHC2, MYD88, p38, and phos-p38. Details of dilution and producers are provided in Table 2.

**Table 2:** Antibodies used for protein analysis.

Antigen	species	Immuno-blot dilution	Cell surface ELISA dilution	producer	Cat-#
C1qR		1:1,00	1:500	Abcam	ab134079
ICAM1		1:500	1:500	Abcam	ab2213
β-defensin		1:1,000	1:500	Abcam	ab14425
MYD88		1:500	1:50	Abcam	ab2068
ICOS		1:1,000	1:100	Abcam	ab133680
ICOSL		1:1,000	1:100	Abcam	ab138354
MHC2		1:500	1:50	Santa Cruz Biotechnology	sc-73601
Erk1/2	Rabbit, pAb	1:2,000	DA	Cell Signalling Technology	9102
phosphorylated Erk1/2	Rabbit, mAb	1:1,000	DA	Cell Signalling Technology	4376
JNK	Rabbit, pAb	1:2,000	DA	Cell Signalling Technology	9252
phosphorylated JNK	Mouse, mAb	1:1,000	DA	Cell Signalling Technology	9255
P38	Rabbit, pAb	1:1,000	DA	Cell Signalling Technology	9212
phosphorylated p38	Mouse, pAb	1:500	DA	Cell Signalling Technology	9216
CREB	Rabbit, mAb	1:1,000	DA	Cell Signalling Technology	4820
phosphorylated CREB	Rabbit, mAb	1:1,000	DA	Cell Signalling Technology	9198

Antibodies used for protein analysis. DA: does not apply, mAb: monoclonal antibody, pAb: poly clonal antibody; Abcam, Cambridge, U.K.; Cell Signalling Technology; Santa Cruz Biotechnology, Santa Cruz, CA, U.S.A.

Membranes were blocked for 1 hour (room temperature) in PBS containing 0.01% Tween-20 and 2% bovine serum albumin. The primary antibodies were added at concentrations indicated in Table 2 and incubated overnight at 4°C. Following 3 washes with blocking buffer, membranes were incubated with secondary species specific antibodies labelled with horse radish peroxidase for 1 hour. Unbound antibody was washed off by 3 washes with blocking buffer and protein bands were visualised by exposure to X-ray films.

### Cell surface specific ELISAs

These analyses were based on the in-house developed ELISA systems for deposition of extracellular matrix molecules [34]. In brief, BEC were seeded into 96-well plate and grown to confluent. Cells were either infected with RV, or OM-85 or signal transduction inhibitors for Erk1/2 MAPK, p38 MAPK or cAMP, alone or in combination as described earlier [34]. BEC were fixed after various incubation periods with 2% formalin in PBS (4°C, 2x5 min). Unspecific binding of antibodies was blocked by 30 min incubation of the fixed cells in 2% bovine serum albumin, in PBS + 0.01% Tween-20. The first antibody specific against one of the cell membrane proteins (Table 2) was added to the blocking buffer and incubated over night at 4°C. Unbound antibody was washed off 3 times with blocking buffer before the secondary antibody was added and incubated for 1 hour at room temperature. Antibody binding was quantitated after 3 washes with blocking buffer by the horse radish peroxidase substrates (TBM). Optical density was determined by ELISA plate reader (Biorad) and changes of antibody binding were calculated as percentage of unstimulated cells.

### IFN-γ ELISA

Secreted IFN-γ was detected by commercial available ELISA kit (R&D Systems, UK) in the cell culture medium of primary human BEC before and after infection with RV at 24, 48 and 72 hours. The ELISA was performed according to instructions from the supplier.

### BEC

BEC isolation and characterisation has been published earlier [34]. Small pieces of bronchial tissues were placed into cell culture vessels, which were pre-wetted with BEC specific medium Cnt-PR-A (CellnTech, Bern, Switzerland). The medium was replaced every second day and cells were passaged by mechanical shaking of dividing cells. Cells were characterised by positive staining of E-Cadherin and Pan-Keratin, and negative staining for fibronectin (supplement Fig 1B).

### Statistics

The Null hypothesis was that OM-85 does not affect RV infection, or the expression cell membrane proteins, or intracellular signalling proteins. Student’s t-test (paired, two-sided) and Wilcoxon test were used for statistical analysis. P-values < 0.05 were considered significant.

## Results

### OM-85 reduced RV infection and RV-induced cell death

**Fig 2:**
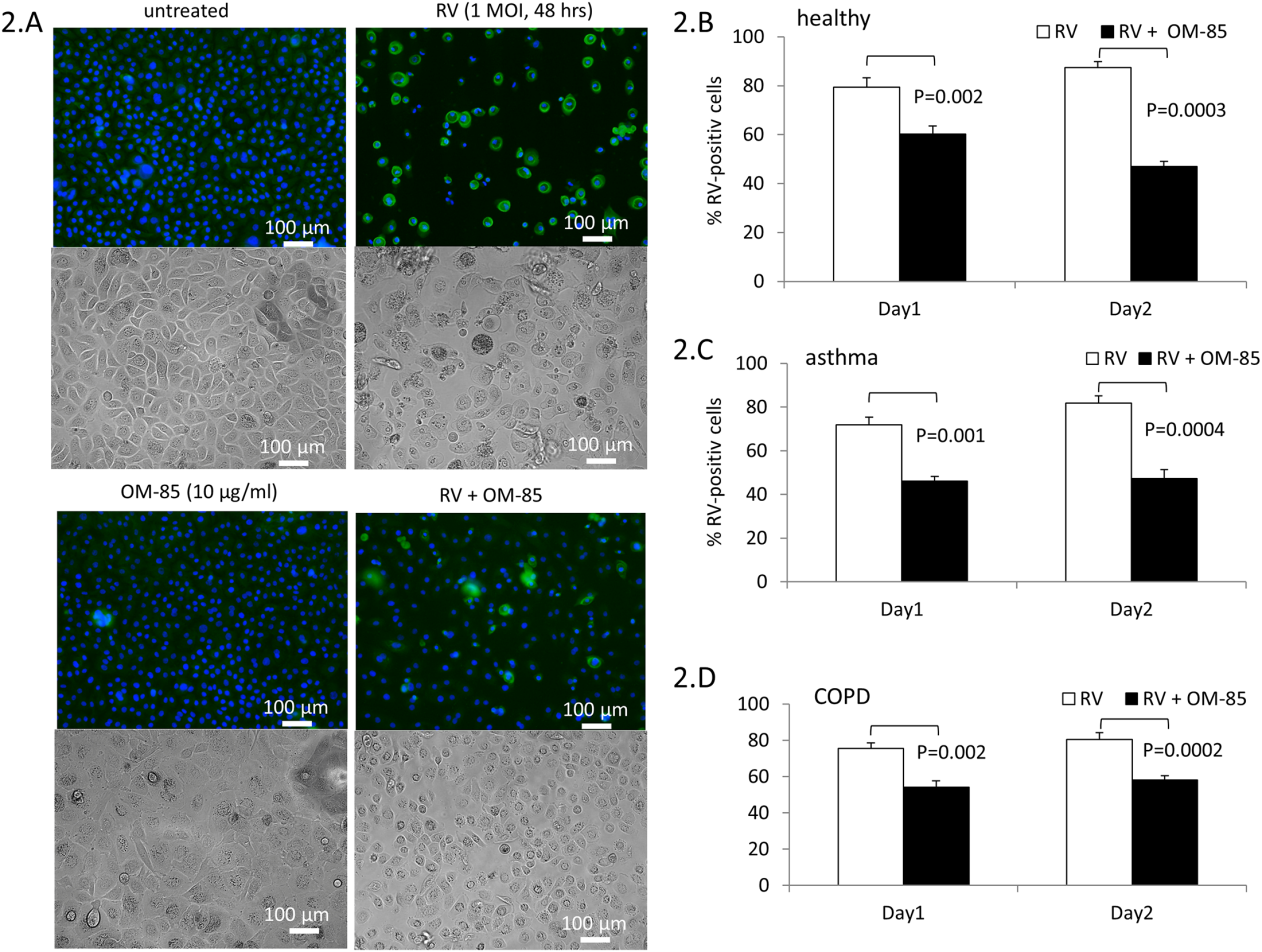
(A) Representative IF images for RV infection (green) in primary BEC; nuclei were stained for cell counting by EVOS live cell staining kit (Thermofisher Scientific). Light microscopic images are depicted for the effect of OM-85 and RV infection on BEC phenotype. (B-D) Quantitation of RV infection of BEC by IF. BEC were pretreated for 24 or 48 hrs with OM-85 (10 μg/ml). Bars represent mean±SEM of RV positive BEC derived from: non-asthma, non-COPD controls (n = 8), (C) COPD (n = 10) and (D) asthma (n = 9). Statistics were performed with paired Student’s t-test, two-sided.

BEC were pre-treated with OM-85 at various concentrations (0.1, 1, 10 μg/ml) and infected with 1 MOI of RV. The number of RV infected cells was detected by immuno-fluorescence (IF) as depicted in Fig 2A. In addition, we show the effect of OM-85 and RV infection on the morphology of primary BEC by light microscopy (Fig 2A). The preventive effect of OM-85 pre-incubation on RV infection of BEC became significant when the cells had been pre-incubated with OM-85 for 48 hrs (Fig 2B–2D). The beneficial effect of OM-85 was detectable in all three cell donor groups with not significant difference comparing the groups to each other. The results used to create Fig 2 are available in online-data pdf.

**Fig 3:**
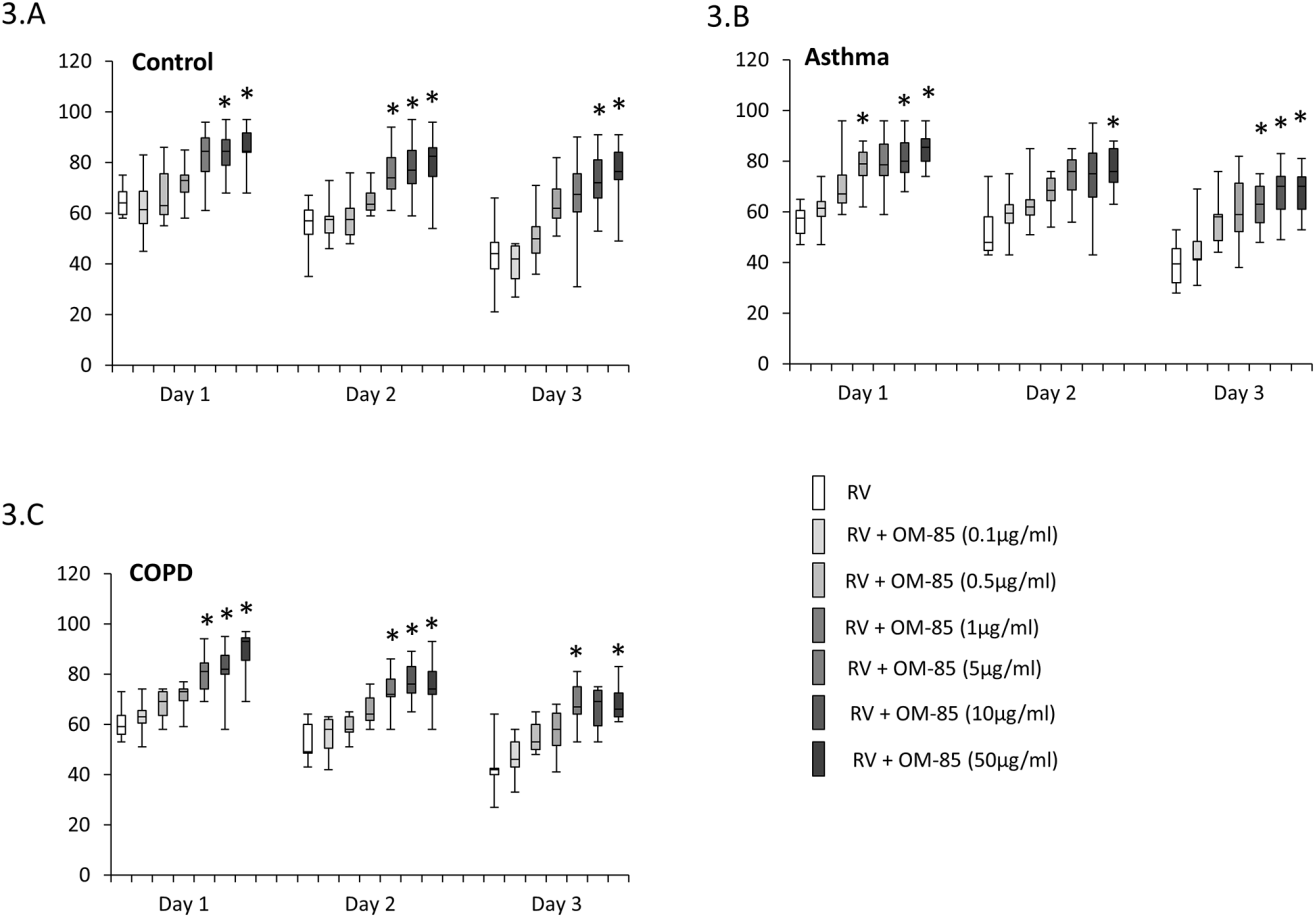
Dose-dependent preventive effect of OM-85 pre-incubation (2 days) on RV-induced cell death as determined by direct cell counting with Trypan blue exclusion staining. Box-plots represent median and 95 % confidence intervals for BEC derived from: (A) non-asthma, non-COPD controls (n = 8), (B) asthma (n = 9) and (C) COPD (n = 10). Statistics were performed with paired Student’s t-test, two-sided.

RV infection of primary BEC decreased cell survival in a time dependent manner, with no significant difference comparing the effect in BEC obtained from controls (Fig 3A), asthma (Fig 3B) or COPD patients (Fig 3C). The survival of BEC after RV infection decreased over three days. After one day, approximately 60% of BEC survived which decreased to 50% after 2 days and further declined to 40% after 3 days, with no significant difference comparing the three donor groups to each other. When the cells have been pre-treated with OM-85 for 24 hours prior to RV infection, the survival in all three groups significantly increased in a dose dependent manner (Fig 3). The results used to create Fig 3 are available in online-data pdf.

### OM-85 activates Erk1/2 MAPK and cAMP signalling in BEC

**Fig 4:**
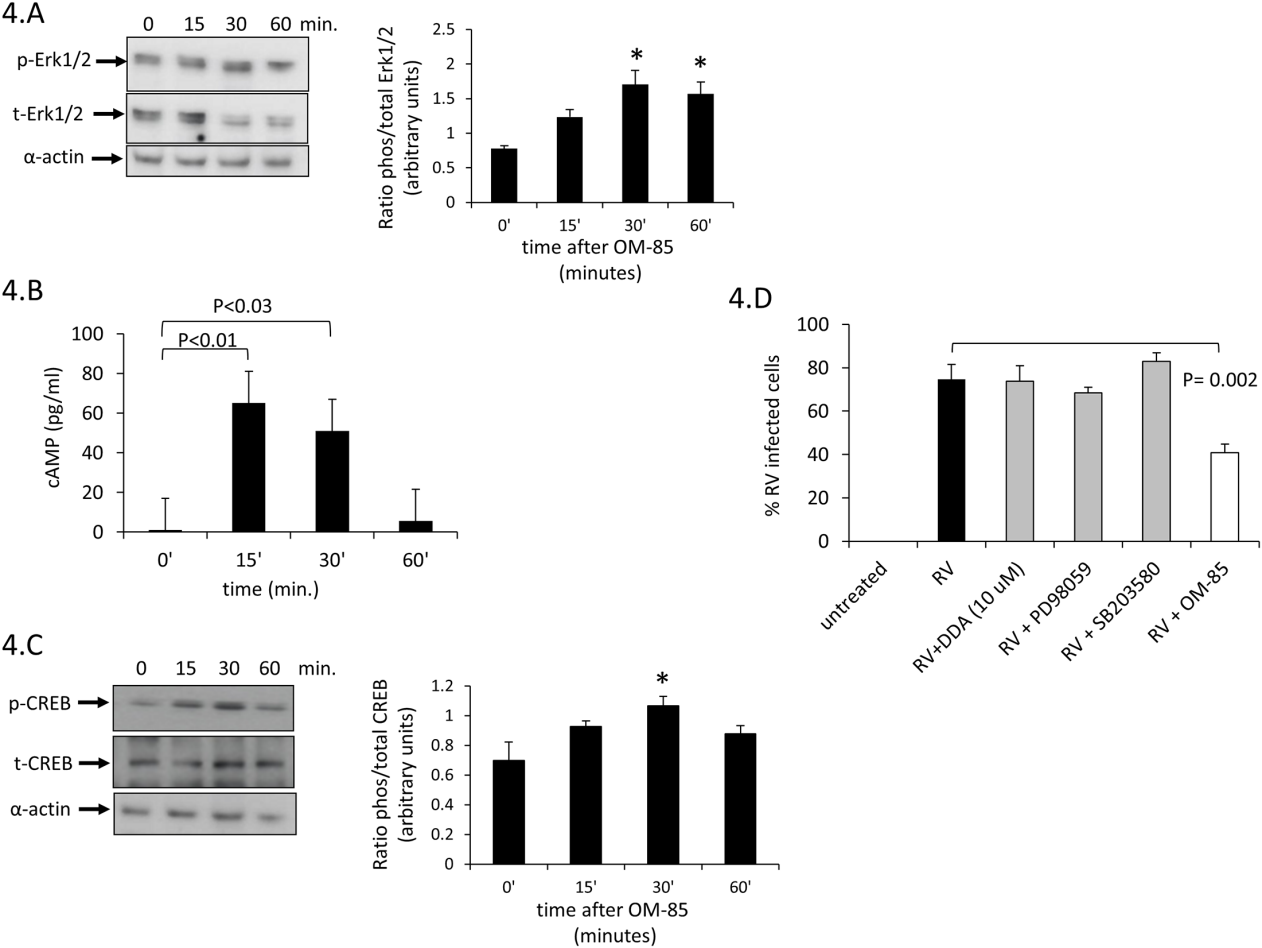
OM-85 induced various cell signalling. (A) Representative Western-blot of the ratio of total and phosphorylated Erk1/2 MAPK and bar chart analysis based on three additional Western-blots in BEC. (B) OM-85 induced activation of intracellular cAMP determined by ELISA in 5 BEC lines. Bars represent mean ± S.E.M. and asterix indicate significant difference compared to non-stimulated BEC. (C) Representative Western-blot of the ratio of total and phosphorylated CREB. Bar chart analysis based on three additional Western-blots in BEC. (D) RV infection was determined by IF and was not affected by inhibition of Erk1/2, p38 MAPK or cAMP (n = 5).

In order to understand which signalling pathways are activated by OM-85 in primary BEC, we exposed confluent cell layers for various time periods (0, 15, 30, 60 minutes) to OM-85 (10 μg/ml). We assessed the effect of OM-85 on all three MAPK pathways (Erk1/2, p38, JNK) but only Erk1/2 MAPK was slowly, but significantly activated by OM-85 at 30 and 60 minutes (Fig 4A) as previously demonstrated on human primary dendritic cells [22].

OM-85 activated the formation of intracellular cAMP significantly within 15 minutes, and sub-sequent decline, we observed no difference comparing the three diagnostic groups (Fig 4B). cAMP activation was followed by an increase of CREBP phosphorylation as determined by immune-blotting (Fig 4C). This finding is in line with the earlier described high basal activity of Erk1/2 MAPK in asthmatic airway smooth muscle cells [35].

In order to exclude any effect of signal transduction inhibitors on the susceptibility of BEC to RV infection, cells were pre-treated with either DDA, SB203580, PD98059 or OM-85 for 30 minutes prior to infection and the cells were grown for additional 24 hours. RV infection was not affected by inhibition of Erk1/2, p38 MAPK or cAMP, while OM-85 had reducing effect (Fig 4D). The results used to create Fig 4 are available in online-data pdf.

### OM-85 increases antigen presenting cell surface proteins

**Fig 5:**
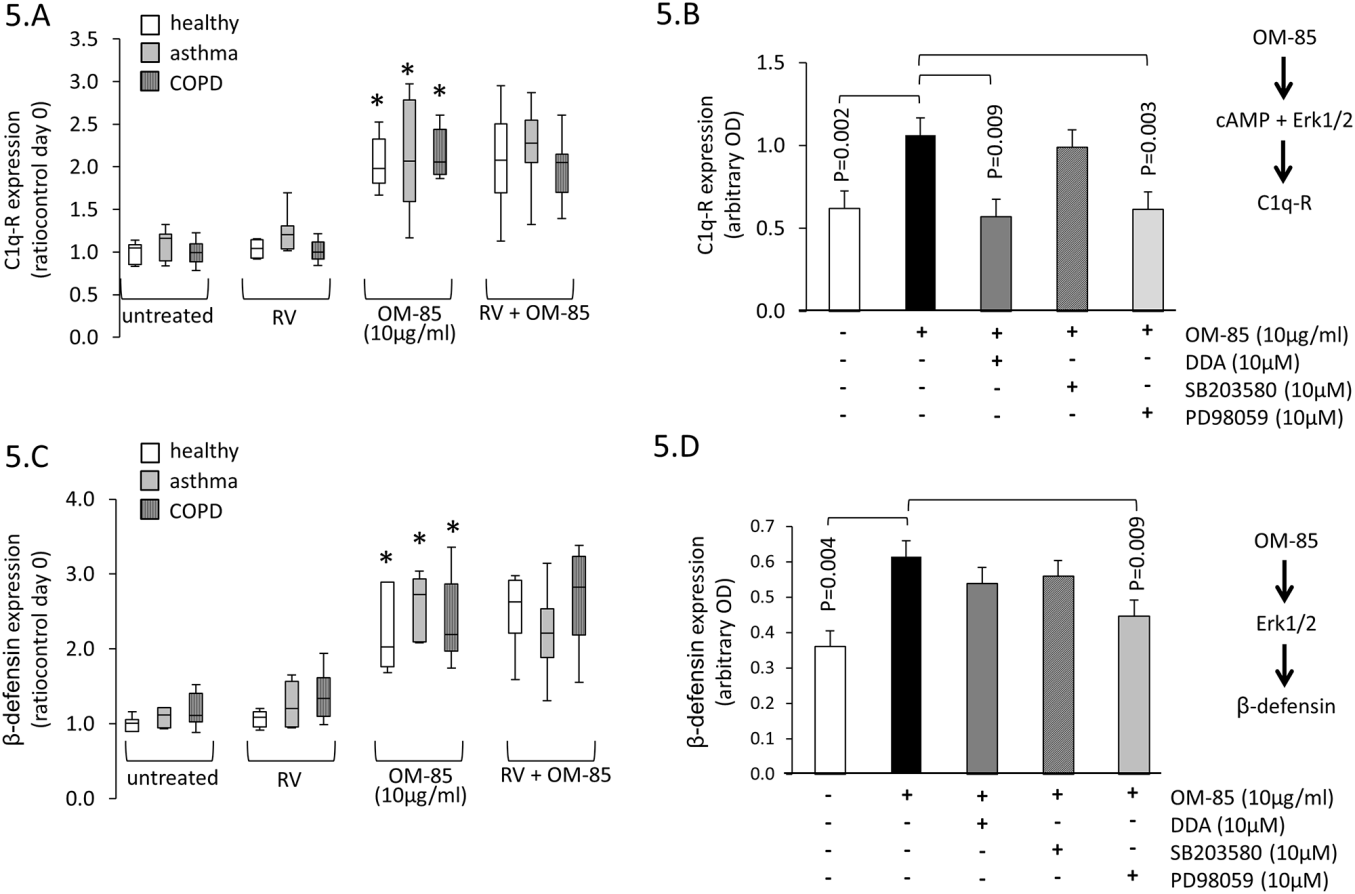
Differential regulation of cell surface RV binding proteins by OM-85 and RV infection. (A) Expression of C1q-R after 48 hrs incubation with OM-85 (10 μg/ml). Box-plots represent median and 95% confidence interval for BEC derived from: non-asthma, non-COPD controls (n = 6), asthma (n = 10) and for COPD (n = 7). (B) shows the effect of cell signal inhibitors (DDA for cAMP; SB203580 for p38 MAPK and PD98059 for Erk1/2 MAPK) on C1q-R expression induced by OM-85. The analysis was performed in 5 BEC lines across all three patient groups. (C) Expression of β-defensin after 48 hrs incubation with OM-85 (10 μg/ml). Box-plots represent median and 95% confidence interval determined in BEC of non-asthma, non-COPD controls (n = 6), asthma (n = 10), and COPD (n = 7). (D) shows the effect of cell signal inhibitors (DDA for cAMP; SB203580 for p38 MAPK and PD98059 for Erk1/2 MAPK) on β-defensin expression induced by OM-85. The analysis was performed in 5 BEC lines across all three patient groups. All statistics were performed with paired Student’s t-test, two-sided.

The cell surface expression of C1qR by BEC was not affected by RV expression but was significantly increased in all three groups by OM-85 (10 μg/ml) and not affected by RV infection (Fig 5A). The up-regulation of C1qR was mediated by cAMP since it was prevented when BEC had been pre-treated with DDA and similarly by the Erk1/2 MAPK inhibitor PD98059 (Fig 5B). In all analysis, we did not observed a disease specific effect on OM-85-induced C1qR expression. OM-85 treatment significantly increased the expression of β-defensin, which again, was not affected by RV infection (Fig 5C). As shown in Fig 5D, inhibition of Erk1/2 MAPK significantly reduced β-defensin expression in BEC while inhibition of Erk1/2 MAPK did not achieve significance. The results used to create Fig 5 are available in online-data pdf.

**Fig 6:**
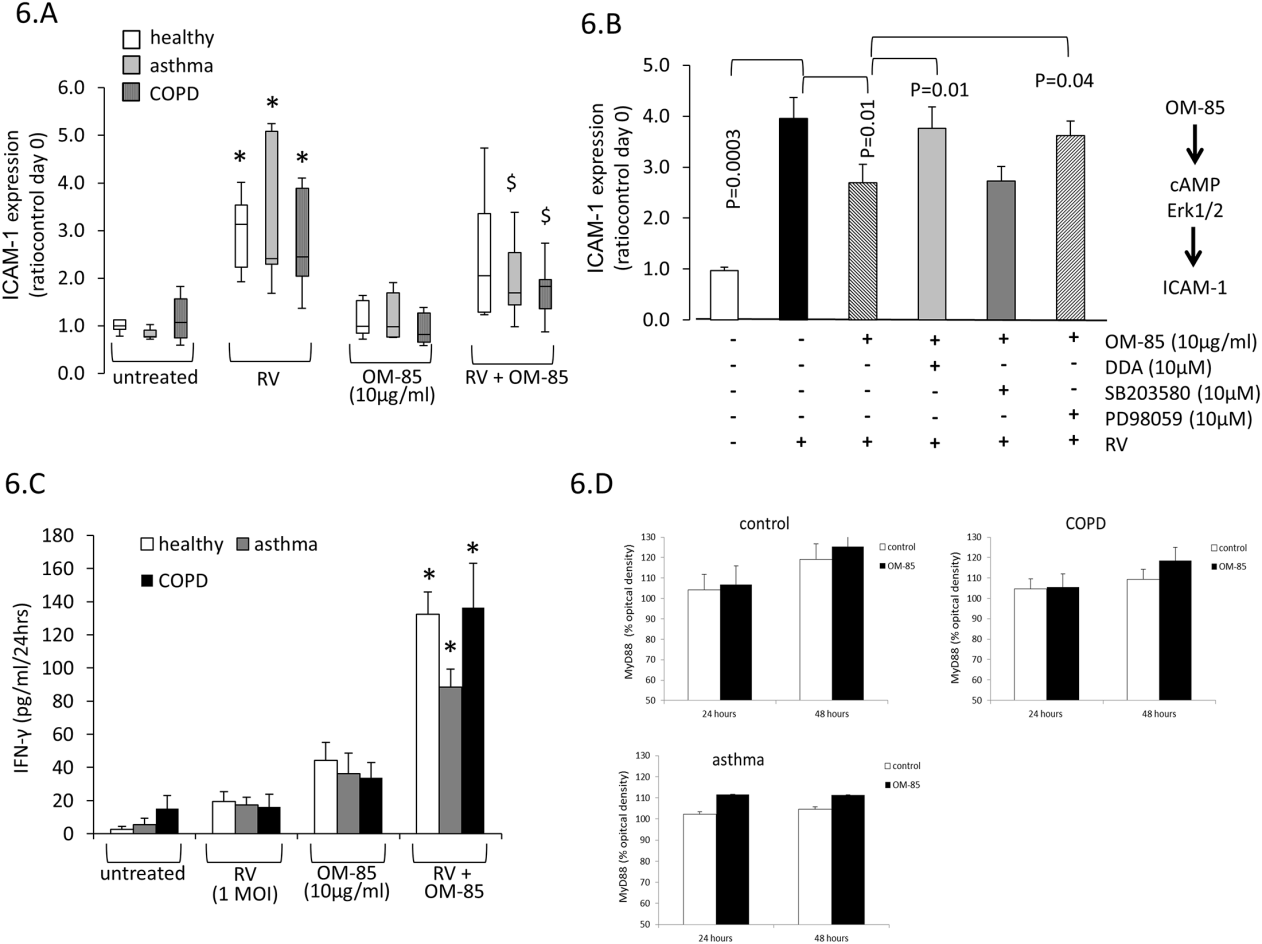
RV induced expression of ICAM-1 on BEC is prevented by OM-85 through cAMP and Erk1/2 MAPK. (A) Increased expression of ICAM-1 by RV and its inhibition by OM-85 pre-incubation (24 hrs). Box-plots represent median and 95% confidence interval of BEC derived from: non-asthma, non-COPD controls (n = 6), asthma (n = 10), and COPD (n = 7). (B) Show the effect of cell signal inhibitors (DDA for cAMP; SB203580 for p38 MAPK and PD98059 for Erk1/2 MAPK) on ICAM-1 expression induced by RV in the presence and absence of OM-85 (10μg/ml). (C) RV and OM-85 induced the secretion of IFN-γ by primary human BEC (n = 5 in each group). (D) OM-85 has a non-significant increasing effect on MyD88 expression by BEC (n = 5 in each group).

In contrast the two previous surface receptors, ICAM1 expression was significantly increased by RV infection, while OM-85 alone had no effect (Fig 6A). However, when the cells were pre-incubated with OM-85 for 48 hours, RV infection dependent expression of ICAM1 was significantly reduced in BEC of asthma and COPD patients, while the effect was not significant in control cells (Fig 6A). Assessing signal transduction, we observed that the inhibition of Erk1/2 MAPK reduced ICAM1 stimulation by RV in BEC of asthma patients and controls but not COPD patient (Fig 6B). In addition, inhibition of cAMP signalling by DDA significantly reduced RV induced ICAM1 expression in asthma and control cells (Fig 6B). When additional signal transduction was inhibited in BEC pre-treated with OM-85 and infected with RV, Erk1/2 MAPK and cAMP prevented the inhibitory effect of OM-85 in all three groups thus confirming that OM-85 signals through these three signalling routes (Fig 6B).

The secretion of IFN-γ by primary human BEC was detected before and after infection and showed a similar increase after 48 hours in all three patient groups (Fig 6C). Pre-incubation with OM-85 further induced the secretion of IFN-γ significantly in cells of all three patient groups (Fig 6C).

OM-85 had no significant effect on the expression of MyD88 by BEC over 48 hours, only a trend of increase expression was observed (Fig 6D). None of the other investigated RV docking proteins (ICOS, ICOSL) were modified by incubation of BEC with OM-85 over 3 days (data not shown). The results used to create Fig 6 are available in online-data pdf.

## Discussions

Considering the cell receptor-mediated maturation and activation induced by OM-85 on myeloid cells (from rodent and human origins) and its anti-viral activity, both demonstrated in vitro and in vivo using various immune myeloid lineage and lung tissue, we have postulated that OM-85 could directly interact with the immune epithelial cell layer from the lung. In the present study, we provide evidence that OM-85 improves cells survival of primary human BEC, which were infected with RV. This beneficial effect was paralleled by the increase of C1q-R and b-defensin, two cell surface proteins known to bind RV and helped killing the virus intracellularly. Furthermore, OM-85 reduces the expression of ICAM1, which has been described to help RV to infect the host’s cells. The regulation of cell surface proteins by OM-85 involves two signalling pathways: Erk1/2 MAPK and cAMP. Together, these results suggest that OM-85 improves hosts defence of human BEC.

In children with re-occurring respiratory tract infections, oral application of OM-85 over cycles of 10 days of 3 consecutive months significantly reduced infections with influenza virus. However, this effect could not be explained through a change in immune globulin levels [16]. A protective effect against respiratory tract infections was also reported in 104 HIV patients who used OM-85 as supplementary therapy [36]. Our finding suggests that OM-85 activates a different line of host’s defence. In animal models, there is sufficient data proving that OM-85 improves recognition, antigen presentation and intracellular killing of viruses in [7, 8, 13, 22]. In mice, OM-85 increased the expression of MHC2, CD86 and CD40 expressions while it decreased the expression of ICOSL; together, these effects seem to increase the production of anti-virus anti-bodies by B-cells [11]. In a murine model for RSV and influenza virus infection, OM-85 has been demonstrated to reduce the infection rate through Toll-like receptors (TLR) signalling, and TLR adaptors Trif and MyD88 [37, 38].

As it had been reported for other viruses, OM-85 significantly improved the survival of human BEC after RV infection. This protective effect of OM-85 was dose and time dependent. The observation that the lytic activity of cell supernatant was reduced indicates that OM-85 either reduced the infection rate of RV and/or improved the intracellular killing of RV. In order to understand the mechanism by which OM-85 reduces RV infection of BEC, we determined the compound effect on RV binding proteins and the underlying intracellular signalling mechanism.

OM-85 has been shown to modify the expression of several membrane proteins in immune cells as well as in epithelial cells and it was suggested that this process involves MAPKs through the TLR, most probably through TLR4 and TLR2-dependend signalling [12, 37]. It was indicated that TLR-membrane proteins respond to OM-85 only when a second adaptor protein MYD88 is present. In line with these earlier results, OM-85 did not up-regulate the expression of MYD88 in BEC. Since there is no specific target of MYD88 the possible modifying effect of OM-85 on the function of MYD88 was not further investigated. Further work to address the involvement of this key adaptor protein in OM-85 signalling could be approached by siRNA or knock-out studies.

The activation of Erk1/2 MAPK by OM-85 stimulated the action of transcription factor NFκB, which increased the secretion of various cytokines in macrophages and it was discussed that the consequence is an activation of the immune response [13]. In dendritic cells of patients with COPD, OM-85 increased the secretion of IL-1α, IL-1β, IL-6 and TNF-α, which was regarded as a strengthening of the immune response during viral infection [20, 22]. Our results on BEC show that OM-85 activates Erk1/2 MAPK but had no effect on p38 or JNK MAPKs, similar results were reported in healthy cells earlier in other cell types [12, 18]. This finding indicates that the activation of Erk1/2 MAPK is a response to OM-85, which is independent of the target cell type.

In BEC, OM-85 did not up-regulate the expression of ICAM1, which is in contrast to previous studies showing stimulatory effect of the substance on ICAM1 in monocytes, granulocytes and dendritic cells [26, 39]. Opposing to these earlier results, ICAM1 expression induced by RV infection in BEC was significantly reduced by OM-85. Thereby, OM-85 may have reduced the docking of RV to BEC in a secondary infection cycle but not for the primary infection. However, overall OM-85 might reduce the infection rate *in vivo* by modulation of ICAM1 expression [40, 41]. These results stress the importance of addressing receptor and cell signalling in each cell type, in particular when the drug is used to target BEC. Accordingly, these results provide for the first time a direct effect on these cells.

BEC expressed β-defensin which helps to clear RV infection and involves the action of IL-17a [41]. In another study, it was indicated that RV infection increased the expression of β-defensin through the activation of TLR3. However, this study determined only the effects on mRNA but not on the protein [42]. In primary BEC, RV had no significant stimulatory effect on β-defensin within the observation period of 3 days, while OM-85 significantly increased its expression through the activation of Erk1/2 MAPK. This effect may further strengthen the protective ability of OM-85 against RV infection of BEC.

In BEC, OM-85 up-regulated the expression of C1qR, which is also known as either calreticulin, surfactant protein receptor, mannan binding ligand receptor, CD93 or Aa4. C1qR is mainly expressed intracellular but also signals apoptosis when expressed on the cell surface [43]. Here it can bind heat shock proteins, integrins as well as viral and bacterial proteins [44]. It has been shown that C1qR response to the presence of viral capsid components as well as to bacterial wall proteins. The activation of C1qR increases the number of B-cells and their secretion of IL-10 [45], this may indicate an anti-inflammatory effect of OM-85. In dendritic cells, the activation of C1qR enhanced the secretion of IFN-γ and the expression of CD40, which both reduced inflammation and combat viral infections [46]. RV infection stimulated the secretion of IFN-γ by primary human BEC with no disease specific effect, suggesting a general anti-viral response. Previous studies demonstrated the capacity of OM-85 to elicit anti-viral responses by stimulating the production of type I IFN [22, 38]. In the present study, RV-induced secretion of IFN-γ was significantly enhanced when the cells were pre-incubated with OM-85, while the substance alone only had a mild effect. It had been described earlier that OM-85 increases the secretion of IFN-γ by immune cells and thereby improves the combat against viral infections [38]. However, the mechanism by which OM-85 stimulates IFN-γ secretion, especially in combination with viral infection remains to be further investigated.

In conclusion, our data demonstrated that OM-85 stimulated anti-viral activities in BEC obtained from all tested probands, including non-diseased, asthma or COPD. The anti-viral activities of OM-85 in BEC were mediated by the selective modulation of various receptors and effector proteins involved in RV infection. Consequently, OM-85 increased the survival of BEC and thereby may benefit the patient’s defense system against RV infection.

## Supporting information

S1 Data Raw data used for analyses to generate Figs 2–6 as described accordingly. (PDF)
