# Supplementary material for: Broncho Vaxom (OM-85) modulates rhinovirus docking proteins on human airway epithelial cells via Erk1/2 mitogen activated protein kinase and cAMP
Source: PLoS One. 2017 Nov 28;12(11):e0188010. doi: 10.1371/journal.pone.0188010 (PMC5705076; doi:10.1371/journal.pone.0188010)
Supplement: S1 Data — (PDF) [file pone.0188010.s001.pdf]

Data

Figure 2: 100 cells were counted based on nucleus staining and within these cells the number of RV positives was counted. The procedure was repeated on the same slide in two randomly selected areas.

RV positive cells of100							
	Day1						
	count 1	count2	mean		count 1	count 2	mean
controls			RV				OM 10
1	81	89	85.0		51.0	58.0	54.5
2	75	68	71.5		53.0	50.0	51.5
3	93	99	96.0		89.0	81.0	85.0
4	79	86	82.5		67.0	76.0	71.5
5	76	75	75.5		65.0	70.0	67.5
6	69	57	63.0		45.0	50.0	47.5
7	95	94	94.5		51.0	55.0	53.0
8	92	99	95.5		47.0	50.0	48.5
9	63	79	71.0		56.0	68.0	62.0
10	64	54	59.0		55.0	52.0	53.5
mean			79.35				59.45
sem			3.78333333				3.21333333
			1				
asthma			RV				OM 10
1	91	96	93.5		44.0	52.0	48.0
2	88	83	85.5		35.0	28.0	31.5
3	66	79	72.5		64.0	57.0	60.5
4	60	65	62.5		45.0	40.0	42.5
5	70	78	74.0		31.0	34.0	32.5
6	69	82	75.5		47.0	45.0	46.0
7	67	60	63.5		56.0	57.0	56.5
8	56	49	52.5		39.0	48.0	43.5
9	54	56	55.0		48.0	53.0	50.5
10	89	80	84.5		54.0	45.0	49.5
mean			71.9				46.1
sem			3.60666667				2.3
COPD			RV				OM 10
1	89	84	86.5		66.0	63.0	66.0
2	54	56	55.0		47.0	56.0	47.0
3	78	71	74.5		55.0	52.0	55.0
4	85	76	80.5		66.0	71.0	66.0
5	81	85	83.0		54.0	63.0	54.0
6	64	65	64.5		42.0	44.0	42.0
7	86	71	78.5		30.0	33.0	30.0
8	80	86	83.0		59.0	68.0	59.0
mean			75.6875				52.375
sem			3.1241126				3.60247388

Figure 3: Epithelial cell survival was manually counted in a modified Neugebauer chamber slide, after trypsin treatment and single cell suspension.

dead cells (%)		RV		
	day	Counts		
control	1	2	3	4
c1	67	59	53	37
c2	73	62	47	26
c3	59	55	41	31
c4	64	51	66	38
c5	58	49	44	42
c6	69	67	37	29
c7	61	54	49	37
c8	58	63	44	28
c9	75	35	21	0
c10	64	59	30	19
mean	64.8	55.4	43.2	28.7
sem	1.931033	2.868217	3.909533	3.847221
asthma	1	2	3	4
a1	65	43	52	38
a2	58	59	41	32
a3	64	61	53	29
a4	61	74	38	21
a5	59	48	32	14
a6	47	44	32	25
a7	49	47	41	27
a8	53	43	28	31
a9	51	48	32	31
a10	57	55	47	41
mean	56.4	52.2	39.6	28.9
sem	1.961859	3.172101	2.793644	2.474088
COPD				
SM1	54	43	39	21
sm2	58	49	43	25
sm3	61	57	41	37
sm4	66	63	64	24
sm5	73	64	42	19
sm6	53	48	42	36
sm7	59	49	27	21
mean	60.57143	53.28571	42.57143	26.14286
sem	2.212518	2.560692	3.458599	2.326862

	OM 0.1			
	day			
control	1	2	3	
c1	68	53	48	3
c2	69	57	44	2
c3	54	58	35	4
c4	55	46	34	2
c5	76	59	48	3
c6	83	73	48	4
c7	45	50	31	3
c8	61	58	41	3
c9	59	52	27	3
c10	62	65	43	3
mean	63.2	57.1	39.9	3
sem	3.527038	2.442449	2.424184	2.21610
asthma	1	2	3	
a1	64	62	41	2
a2	47	69	41	3
a3	58	57	31	2
a4	64	63	54	3
a5	59	47	41	4
a6	51	43	38	2
a7	69	58	49	4
a8	63	61	46	2
a9	60	55	42	2
a10	74	75	69	7
mean	60.9	59	45.2	37.
sem	2.505328	2.992583	3.292416	4.70094
SM1	64	58	42	2
sm2	62	61	58	4
sm3	59	52	46	3
sm4	63	49	44	2
sm5	74	63	52	3
sm6	51	42	33	1
sm7	67	63	54	2
mean	62.85714	55.42857	47	3
sem	2.231805	2.535463	2.664583	2.79284

OM .5

control	day 1	2	3	4
c1	56	58	53	42
c2	74	63	55	39
c3	59	51	54	38
c4	63	48	44	23
c5	55	51	47	31
c6	76	71	68	63
c7	63	59	45	32
c8	86	76	71	54
c9	76	53	41	17
c10	61	57	36	26
mean	66.9	58.7	51.4	36.5
sem	3.281091	2.863758	3.56277	4.435338

asthma	1	2	3	4
a1	75	63	51	38
a2	96	85	76	42
a3	68	51	44	27
a4	66	61	59	32
a5	61	58	59	43
a6	59	53	46	38
a7	86	75	61	41
a8	63	65	48	37
a9	65	61	57	43
a10	73	64	59	32
mean	71.2	63.6	56	37.3
sem	3.723201	3.173501	2.955221	1.713022

COPD				
SM1	69	58	53	28
sm2	73	51	49	32
sm3	58	56	48	25
sm4	73	65	58	33
sm5	61	63	51	28
sm6	66	63	65	31
sm7	74	58	62	28
mean	67.71429	59.14286	55.14286	29.28571
sem	1.997618	1.543033	2.093072	0.889087

OM1

control	day 1	2	3	
c1	75	62	65	3
c2	58	59	51	3
c3	66	61	58	2
c4	72	68	55	4
c5	85	76	82	4
c6	75	68	58	4
c7	85	64	71	4
c8	68	63	59	3
c9	69	71	65	3
c10	74	61	73	4
mean	72.7	65.3	63.7	38.
sem	2.607894	1.686877	2.985335	2.08593

asthma	1	2	3	
a1	69	58	45	2
a2	79	66	53	4
a3	85	75	82	6
a4	79	76	64	3
a5	74	64	54	2
a6	62	54	38	2
a7	75	71	74	6
a8	88	69	73	5
a9	79	68	52	2
a10	85	74	66	5
mean	77.5	67.5	60.1	4
sem	2.495552	2.291288	4.420784	5.16612

SM1	74	76	64	3
sm2	68	61	48	2
sm3	71	64	41	1
sm4	59	62	58	4
sm5	77	73	65	3
sm6	73	58	55	2
sm7	74	68	68	5
mean	70.85714	66	57	33.1428
sem	1.874643	2.089657	3.098387	4.04733

OM10

	day			
control	1	2	3	4
c1	79	74	75	42
c2	86	84	75	36
c3	89	95	91	66
c4	75	64	69	48
c5	97	85	69	43
c6	83	79	84	39
c7	68	71	65	52
c8	79	75	83	48
c9	94	97	64	61
c10	89	59	53	36
mean	83.9	78.3	72.8	47.1
sem	2.810496	3.896009	3.542755	3.216105

asthma	1	2	3	4
a1	96	95	83	41
a2	88	81	75	39
a3	81	84	72	48
a4	79	64	51	30
a5	68	43	49	21
a6	77	71	67	55
a7	85	78	74	53
a8	91	86	74	37
a9	69	61	59	43
a10	75	72	68	32
mean	80.9	73.5	67.2	39.9
sem	2.903829	4.702836	3.476269	3.338163

COPD				
SM1	88	80	65	34
sm2	79	71	54	32
sm3	95	86	75	54
sm4	82	76	69	41
sm5	58	74	53	47
sm6	87	89	74	34
sm7	81	65	73	49
mean	81.42857	77.28571	66.14286	41.57143
sem	3.682649	2.656528	2.93501	2.725541

OM50

	day			
control	1	2	3	
c1	96	96	79	4
c2	84	76	69	3
c3	88	82	85	4
c4	79	74	81	5
c5	68	54	49	2
c6	93	85	86	4
c7	85	94	73	4
c8	84	61	74	3
c9	97	86	91	4
c10	84	83	74	6
mean	85.8	79.1	76.1	43.
sem	2.707192	4.225452	3.704202	3.40408

asthma	1	2	3	
a1	86	78	73	2
a2	89	74	61	4
a3	85	85	53	3
a4	79	63	61	2
a5	83	74	71	4
a6	96	88	75	3
a7	74	71	69	5
a8	79	64	61	3
a9	88	85	81	7
a10	93	87	74	4
mean	85.2	76.9	67.9	41.
sem	2.128119	2.922898	2.709859	4.59516

SM1	86	76	63	2
sm2	97	93	76	5
sm3	93	70	83	3
sm4	69	74	63	5
sm5	85	74	61	3
sm6	96	58	66	4
sm7	93	86	69	2
mean	88.42857	75.85714	68.71429	40.1428
sem	3.076021	3.55166	2.547641	3.44203

Figure 4: Signal transduction of RV and OM-85.

4.A: **intra cellular Erk ½ MAPK expression (optical density) in minutes (T=minutes) after addition of compounds. Statistics were calculated as**

total Erk	0'	15'	30'	60'
a	100596	116543	110694	103725
b	118475	109687	110583	115342
c	109485	110682	128574	108275
d	142356	132534	128574	119857
mean	117728	117361.5	119606.25	111799.75
sem	7325.13154	4379.92348	5177.53288	3348.48722

phos Erk	0'	15'	30'	60'
a	86758	124138	143265	156325
b	99706	132436	154326	117482
c	76850	176574	253418	198675
d	99685	142367	276453	228563
meam	90749.75	143878.75	206865.5	175261.25
sem	5164.83117	9438.30569	33526.7301	22145.8573

ratio phos/total Erk	0'	15'	30'	60'
a	0.862439858	1.06516908	1.29424359	1.50711015
b	0.841578392	1.20739924	1.39556713	1.01855352
c	0.701922638	1.59532715	1.97098947	1.83491111
d	0.700251482	1.07419228	2.150147	1.90696413
mean	0.776548093	1.23552194	1.7027368	1.56688473
sem	0.043567448	0.10386682	0.20659408	0.17554502

4.B: intra cellular cAMP levels (pg/ml) in minutes (T=minutes) after addition of compounds. A=asthma; C=COPD; H=healthy

Cell lines		OM 10µg/ml			
		T0	T15	T30	T60
asthma	A1	0.942	38.942	12.458	2.791
	A2	0.897	93.085	22.714	21.621
	A3	0.947	54.858	41.679	2.341
	mean	0.929	62.295	25.617	8.918
COPD	C1	0.602	193.359	19.474	6.164
	C2	0.615	14.01	5.2715	16.487
	C3	0.704	5.973	38.202	0.859
	C4	0.642	58.485	72.606	0.999
	mean	0.641	67.957	33.888	6.127

healthy	H1	2.916	45.801	47.21	1.233
	H2	1.172	49.94	90.84	1.593
	H3	1.176	95.993	158.76	1.447
	mean	1.755	63.911	98.937	1.424
ALL	MEAN	1.0613	65.0446	50.92145	5.5535

4.C Expression of total and phosphorylated CREB by western-blotting (optical density)

total CREB	0'	15'	30'	60'
a	116253	109584	113256	116453
b	119865	116254	128475	129834
c	128475	127594	123847	128274
d	97856	132860	110968	94564
mean	115612.25	121573	119136.5	117281.25
sem	5125.78786	4996.38923	4055.59697	6797.00038

phos Creb	0'	15'	30'	60'
a	65746	112635	119856	97865
b	59483	110293	109567	109485
c	77512	101928	143527	96857
d	110293	123957	132437	100968
meam	78258.5	112203.25	126346.75	101293.75
sem	9247.5636	3517.65085	6717.61472	2364.6102

ratio phos/total Creb	0'	15'	30'	60'
a	0.56554239	1.02784166	1.05827506	0.84038196
b	0.49624995	0.94872434	0.8528274	0.8432691
c	0.6033236	0.79884634	1.15890575	0.75507897
d	1.12709491	0.93298961	1.19347019	1.06772133
mean	0.69805272	0.92710049	1.0658696	0.87661284
sem	0.12385381	0.03702378	0.06369234	0.05516827

4.D: Effect of signal inhibition on RV replication as described in figure 2.

	untreated	RV	RV+DDA (10 uM)	RV + PD98059	RV + SB203580	RV + OM-85
E1	0	95	95	74	69	43
E2	0	52	86	72	85	25
E3	0	85	63	69	93	51
E4	0	63	76	72	91	37
E5	0	78	49	55	76	48
mean	0	74.6	73.8	68.4	82.8	40.8
sem	0	6.84	7.12	2.68	4.12	3.92

Figure 5: Role of CqR1 and β-defensin in RV and OM-85 signalling assessed by western-blot densitometry.

A. C1qR expression determined by Western-blotting (optical density)

control	day 0	RV day 2	OM 10 ug/ml	RV + OM 10 ug
control				
c1	1.098	1.132	2.657	2.473
c2	0.905	1.218	2.186	2.196
c3	1.125	0.968	1.978	1.978
mean	1.042667	1.106	2.273667	2.215667
sem	0.069273	0.07333	0.200852	0.143232
asthma				
a1	1.165	0.978	1.978	2.318
a2	1.121	1.263	2.687	2.456
a3	0.867	1.177	1.994	1.978
mean	1.051	1.139333	2.219667	2.250667
sem	0.092873	0.084401	0.233712	0.142034
COPD				
sm1	1.005	0.978	2.756	2.758
sm2	0.954	0.894	2.186	1.473
sm3	1.102	1.123	2.574	2.175
mean	1.020333	0.998333	2.505333	2.135333
sem	0.043406	0.066884	0.168089	0.371477

B. Signalling control of C1qR expression determined by Western-blotting (optical density)

Cell lines	Control	OM-85	OM+DDA	OM+SB	OM+PD
H01	0.783	1.251	0.824	1.159	0.686
H02	0.736	1.152	0.663	1.143	0.632
H03	0.718	0.834	0.652	0.997	0.569
H04	0.476	1.135	0.345	0.75	0.742
H05	0.327	0.718	0.516	1.155	0.506
H06	0.686	1.282	0.418	0.737	0.553
mean	0.62	1.06	0.57	0.99	0.61
SD	0.18	0.23	0.18	0.20	0.09
SEM	0.08014088	0.10351232	0.07912859	0.08963322	0.03964812

C β-defensin expression determined by western-blotting (optical density)

defensin beta

control	day 0	RV day 2	OM 10 ug/ml	RV + OM 10 ug	control	day 0	RV day 2	OM 10 ug/ml	RV [illegible] ug
control					control				

h1	95598	122330	308586	317304	h1	0.897	1.147	2.895
h2	108867	103443	191196	169790	h2	1.021	0.970	1.793
h3	108383	113613	219187	266938	h3	1.017	1.066	2.056
h4	124074	118069	308586	293293	h4	1.164	1.108	2.895
h5	95598	97438	212698	308586	h5	0.897	0.914	1.995
h6	107124	128529	179476	257349	h6	1.005	1.206	1.684
mean	106607				mean	1.000	1.068	2.220
					sem	0.057	0.063	0.312
asthma					asthma			
a1	99412	167211	223219	235620	a1	0.933	1.568	2.094
a2	119436	118318	290408	335133	a2	1.120	1.110	2.724
a3	129500	102258	323851	223321	a3	1.215	0.959	3.038
a4	121673	128483	312974	270079	a4	1.141	1.205	2.936
a5	110186	101241	222203	139461	a5	1.034	0.950	2.084
a6	100936	176461	291425	249647	a6	0.947	1.655	2.734
a7	129601	136412	261642	201060	a7	1.216	1.280	2.454
mean	115821				mean	1.086	1.247	2.581
					sem	0.068	0.160	0.221
COPD					COPD			
c1	125825	154913	325385	310728	c1	1.180	1.453	3.052
c2	137212	105756	245335	309826	c2	1.287	0.992	2.301
c3	109138	112295	185693	165962	c3	1.024	1.053	1.742
c4	94368	132589	220869	225041	c4	0.885	1.244	2.072
c5	154688	136535	206663	292914	c5	1.451	1.281	1.939
c6	112408	178590	357969	257625	c6	1.054	1.675	3.358
c7	162692	149727	247703	360449	c7	1.526	1.404	2.324
c8	110942	207114	223012	356728	c8	1.041	1.943	2.092
mean	125909				mean	1.181	1.381	2.360
					sem	0.129	0.182	0.323

D Signalling control β-defensin expression determined by western-blotting (optical density

Cell lines	Control	OM	OM+DDA	OM+SB	OM+PD
H01	0.4	0.595	0.755	0.548	0.5
H02	0.424	0.839	0.481	0.757	0.486
H03	0.218	0.602	0.789	0.557	0.417
H04	0.434	0.601	0.492	0.532	0.465
H05	0.254	0.499	0.416	0.453	0.335
H06	0.434	0.549	0.3	0.509	0.477
mean	0.36	0.61	0.54	0.56	0.45
SD	0.10	0.12	0.19	0.10	0.06
SEM	0.16129504	0.27466368	0.24097359	0.25014147	0.19975541

Figure 6: The effect of OM-85 and RV infection on Icam, INF-γ and Myd88 expression.

A. ICAM expression was detected by Western-blotting (optical density)

Icam 1

control	day 0	RV day 2	OM 10 ug/ml	RV + OM 10 ug
control				
h1	184297	657495	160143	352424
h2	183304	534258	145419	214231
h3	160143	382758	243689	319285
h4	128374	490852	268835	474308
h5	166430	315243	117564	201764
h6	160143	553452	164444	775072
mean	163781.8			
asthma				
a1	139351	832819	159710	277691
a2	127465	584719	236088	415904
a3	118613	375313	276426	402879
a4	125315	275162	125947	161228
a5	148582	395419	160975	236341
a6	167930	388084	312971	276426
a7	126959	859627	123671	554496
mean	136316.3			
COPD				
c1	217264	663520	95553	447612
c2	129679	446390	165333	325573
c3	228347	558548	101462	223704
c4	298674	334742	227164	312645
c5	134875	223806	124178	142537
c6	119594	335264	222686	285764
c7	266453	671928	129781	221667
c8	96572	355013	137727	314253
mean	186432			

control	day 0	RV day 2	OM 10 ug/ml	RV ug
control				
h1	1.125258	4.014458	0.977783	
h2	1.119198	3.262011	0.887883	
h3	0.977783	2.337	1.487887	
h4	0.783811	2.996985	1.641423	
h5	1.016167	1.924775	0.717809	
h6	0.977783	3.379204	1.004046	
mean	1	2.985739	1.119472	
sem	0.072057	0.434821	0.209209	
asthma				
a1	0.850835	5.084934	0.97514	
a2	0.778259	3.570108	1.441478	
a3	0.724213	2.29154	1.687772	
a4	0.765133	1.680051	0.768994	
a5	0.907197	2.414301	0.982861	
a6	1.025325	2.36952	1.910904	
a7	0.775171	5.248615	0.755096	
mean	0.832305	3.23701	1.217463	
sem	0.060365	0.827303	0.26676	
COPD				
c1	1.326546	4.051244	0.583417	
c2	0.791781	2.725517	1.009474	
c3	1.394215	3.410317	0.619492	
c4	1.823609	2.043826	1.386992	
c5	0.823502	1.36649	0.758194	
c6	0.730205	2.047016	1.359648	
c7	1.626878	4.10258	0.792403	
c8	0.589637	2.167601	0.840917	
mean	1.138297	2.739324	0.918817	
sd	0.462298	1.014507	0.309764	
sem	0.266908	0.585726	0.178842	

B. He role of intracellular signalling on ICAM expression

Cell lines	Control	RV	RV+ OM	RV+ OM+DDA	RV1+ OM+SB	RV+ OM+PD
H01	1.174	4.386	2.968	4.233	1.978	3.879
H02	0.886	3.975	3.175	3.996	2.735	4.285
H03	1.047	4.823	3.867	4.175	2.186	3.576

H04	0.978	3.968	1.685	2.286	3.296	3.773
H05	0.737	2.175	1.953	3.004	3.191	2.396
H06	1.006	4.396	2.504	4.867	2.968	3.818
mean	0.97	3.95	2.69	3.76	2.73	3.62
SEM	0.06640462	0.41485548	0.3625247	0.42053827	0.28789045	0.28789045

C. Interferon-γ (pg/ml/24 hours) determined by ELISA

Date/Time 08/29/2017 07:39:35

Quadratic Fit: Y=A+BX+CX^2

20/50/80%: X = 99.458 / 279.762 / 529.291 Y = 0.476 / 1.111 / 1.746

A: 0.063 (+/-0.033), B: 0.004 (+/-0.000), C: -0.000 (+/-0.000)

chi2=0.060, RMS=0.074, r^2=0.992

Standards Report:

Std #	Conc	Well	Replicates	Mean	SD	%CV
1	15	G2	0.049	0.053	0.006	10.673
		H2	0.057			
2	31	E2	0.21	0.2	0.013	6.701
		F2	0.191			
3	62	C2	0.472	0.464	0.011	2.438
		D2	0.456			
4	125	A2	0.528	0.538	0.013	2.5
		B2	0.547			
5	250	G1	0.96	0.962	0.002	0.221
		H1	0.963			
6	500	E1	1.738	1.716	0.031	1.813
		F1	1.694			
7	1000	C1	2.174	2.17	0.006	0.293

Sample Report:

Sample ID	Well	Replicates	Mean	Conc	
A1-C	E9	0.066	0.066	0.728	(*)
A1-OM	G9	0.384	0.384	76.291	(*)
A1-RV	F9	0.193	0.193	30.172	(*)
A1-RV-OM	H9	0.539	0.539	115.557	(*)
A2-RV-OM	D10	0.417	0.417	84.504	(*)
A2-C	A10	0.06	0.06	(-)	(*)
A2-OM	C10	0.101	0.101	8.752	(*)
A2-RV	B10	0.108	0.108	10.365	(*)
A3-C	E10	0.081	0.081	4.159	(*)
A3-OM	G10	0.088	0.088	5.764	(*)
A3-RV	F10	0.145	0.145	18.936	(*)
A3-RV-OM	H10	0.477	0.477	99.636	(*)
A4-C	A11	0.03	0.03	(-)	(*)
A4-OM	C11	0.273	0.273	49.206	(*)
A4-RV	B11	0.085	0.085	5.076	(*)
A4-RV-OM	D11	0.298	0.298	55.236	(*)

A5-C	E11	0.175	0.175	25.943	(*)
A5-OM	G11	0.374	0.374	73.817	(*)
A5-RV	F11	0.188	0.188	28.995	(*)
A5-RV-OM	H11	0.485	0.485	101.674	(*)
A6-C	A12	0.04	0.04	(-)	(*)
A6-OM	C12	0.109	0.109	10.596	(*)
A6-RV	B12	0.078	0.078	3.472	(*)
A6-RV-OM	D12	0.278	0.278	50.409	(*)
A7-C	E12	0.094	0.094	7.142	(*)
A7-OM	G12	0.189	0.189	29.231	(*)
A7-RV	F12	0.211	0.211	34.421	(*)
A7-RV-OM	H12	0.526	0.526	112.194	(*)
C1-C	A6	0.027	0.027	(-)	(*)
C1-OM	C6	0.094	0.094	7.142	(*)
C1-RV	B6	0.078	0.078	3.472	(*)
C1-RV-OM	D6	0.311	0.311	58.387	(*)
C2-C	E6	0.029	0.029	(-)	(*)
C2-OM	G6	0.211	0.211	34.421	(*)
C2-RV	F6	0.069	0.069	1.413	(*)
C2-RV-OM	H6	0.738	0.738	168.806	(*)
C3-C	A7	0.316	0.316	59.602	(*)
C3-OM	C7	0.177	0.177	26.412	(*)
C3-RV	B7	0.166	0.166	23.835	(*)
C3-RV-OM	D7	0.738	0.738	168.806	(*)
C4-C	E7	0.118	0.118	12.674	(*)
C4-OM	G7	0.145	0.145	18.936	(*)
C4-RV	F7	0.167	0.167	24.069	(*)
C4-RV-OM	H7	0.434	0.434	88.765	(*)
C5-C	A8	0.263	0.263	46.805	(*)
C5-OM	C8	0.383	0.383	76.043	(*)
C5-RV	B8	0.288	0.288	52.819	(*)
C5-RV-OM	D8	0.998	0.998	244.287	(*)
C6-C	E8	0.077	0.077	3.243	(*)
C6-OM	G8	0.176	0.176	26.177	(*)
C6-RV	F8	0.041	0.041	(-)	(*)
C6-RV-OM	H8	0.384	0.384	76.291	(*)
C7-C	A9	0.188	0.188	28.995	(*)
C7-OM	C9	0.263	0.263	46.805	(*)
C7-RV	B9	0.094	0.094	7.142	(*)
C7-RV-OM	D9	0.665	0.665	148.868	(*)
H1-C	A3	0.039	0.039	(-)	(*)
H1-OM	C3	0.184	0.184	28.055	(*)
H1-OM-RV	D3	0.637	0.637	141.349	(*)
H1-RV	B3	0.176	0.176	26.177	(*)
H2-C	E3	0.059	0.059	(-)	(*)
H2-OM	G3	0.265	0.265	47.285	(*)
H2-RV	F3	0.095	0.095	7.372	(*)

H2-RV-OM	H3	0.639	0.639	141.884	(*)
H3-C	A4	0.051	0.051	(-)	(*)
H3-OM	C4	0.364	0.364	71.35	(*)
H3-RV	B4	0.202	0.202	32.294	(*)
H3-RV-OM	D4	0.738	0.738	168.806	(*)
H4-C	E4	0.105	0.105	9.673	(*)
H4-OM	G4	0.189	0.189	29.231	(*)
H4-RV	F4	0.208	0.208	33.711	(*)
H4-RV-OM	H4	0.531	0.531	113.486	(*)
H5-C	A5	0.045	0.045	(-)	(*)
H5-OM	C5	0.134	0.134	16.38	(*)
H5-RV	B5	0.057	0.057	(-)	(*)
H5-RV-OM	D5	0.656	0.656	146.444	(*)
H6-C	E5	0.089	0.089	5.993	(*)
H6-OM	G5	0.374	0.374	73.817	(*)
H6-RV	F5	0.134	0.134	16.38	(*)
H6-RV-OM	H5	0.407	0.407	82.007	(*)

Analysis:

Healthy	untreated	RV	OM	RV+OM
H1	0	26.177	28.055	141.349
H2	0	7.372	47.285	141.884
H3	0	32.294	71.35	168.806
H4	9.673	33.711	29.231	113.486
H5	0	0	16.38	146.444
H6	5.993	16.38	73.817	82.007
Mean	2.611	19.3223333	44.353	132.329333
SEM	1.8823289	6.15501317	10.737505	13.5496502
Asthma				
A1	0.728	30.172	76.291	115.557
A2	0	10.365	8.752	84.504
A3	4.159	18.936	5.764	99.636
A4	0	5.076	49.206	55.236
A5	25.943	28.995	73.817	101.674
A6	0	3.472	10.596	50.409
A7	7.143	24.421	29.231	112.194
Mean	5.42471429	17.3481429	36.2367143	88.4585714
SEM	3.85823897	4.54778428	12.444709	10.7553935
COPD				
C1	0	3.472	7.142	58.387
C2	0	1.413	34.421	168.806
C3	59.6.2	23.835	26.412	168.806
C4	12.674	24.069	18.936	88.765
C5	46.805	52.819	76.043	244.287

C6	3.243	0	26.177	76.291
C7	28.995	7.142	46.805	148.868
Mean	15.2861667	16.1071429	33.7051429	136.315714
SEM	7.74718019	7.79289189	9.12597615	26.8035637

D. MyD88 expression and modification was determined by an in house developed ELISA

Myd88	CONTROLS				
		24 hrs		48 hrs	
control		control 24	OM 10 ug/ml	control 48	OM 10 ug
control	day0				
h1	165473	184297	160143	198574	203752
h2	142537	132464	149968	176453	214231
h3	156342	160143	176453	143527	177564
h4	114967	128374	142561	142364	154928
h5	152436	166430	164735	198672	186252
h6	169584	160143	164444	217684	221761
mean	150223.167	155308.412	159717.399	179545.667	193081.333

Analysis controls: ratio day 0 / 24 / 48 hrs

	24 hrs		48 hrs	
control	control 24	OM 10 ug/ml	control 48	OM 10 ug
control				
h1	111.375764	96.7789404	120.003868	123.133079
h2	92.933063	105.213383	123.794524	150.298519
h3	102.431219	112.863466	91.8032263	113.574088
h4	111.661607	124.00167	123.830317	134.75867
h5	109.179998	108.068304	130.331418	122.183736
h6	94.4328569	96.9692766	128.363525	130.767643
mean	103.669085	107.31584	119.687813	129.119289
sem	4.86994777	5.95682932	8.1659038	7.34243084

COPD

		24 hrs		48 hrs	
l		control 24	OM 10 ug/ml	control 48	OM 10 ug
l	day0				
c1	198675	217264	195553	210697	226175
c2	110895	109679	165333	142365	152438
c3	216643	228347	201462	238107	217564
c4	178539	198674	127164	197236	210985
c5	110978	114875	124178	132978	148275
c6	109685	129594	142686	123351	130075

c7	164739	176453	129781	168664	187564
c8	109786	96572	137727	126328	132467
mean	149993	158932	152985	167466	175693

Analysis COPD: ratio day 0 / 24 / 48 hrs

	24 hrs		48 hrs	
	control 24	OM 10 ug/ml	control 48	OM 10 ug
c4	111.277648	71.2247744	110.472222	118.17306
c5	103.511107	111.89453	119.823749	133.607562
c6	118.151257	130.086734	112.459315	118.589597
c7	107.110642	78.7798311	102.382557	113.85525
c8	87.9636857	125.45032	115.067495	120.659283
mean	105.209618	107.243408	113.067771	119.576642
sem	5.18801988	15.4525235	4.70335748	6.7374506

Asthma

		24 hrs		48 hrs	
l		control 24	OM 10 ug/ml	control 48	OM 10 ug
l	day0				
a1	117236	120967	138376	126385	137691
a2	132454	127465	142309	140680	135904
a3	109685	118613	139935	118734	142879
a4	117587	117163	125947	124231	121228
a5	130895	139286	145398	129786	136341
a6	164538	167930	153648	170065	176426
a7	110986	126959	134256	112453	154496
mean	126197.286	131197.419	139981.313	131762	143566.408

Analysis asthma: ratio day 0 / 24 / 48 hrs

	24 hrs		48 hrs	
	control 24	OM 10 ug/ml	control 48	OM 10 ug
a3	108.139594	127.578976	108.249989	130.263261
a4	99.6394159	107.109789	105.650284	103.096069
a5	106.410482	111.079873	99.152756	104.160325
a6	102.061277	93.3814681	103.359102	107.225236
a7	114.391736	120.966608	101.321788	139.203508
mean	104.294014	112.227005	104.535471	114.857221
sem	3.44879821	6.46069367	1.96092367	8.49156211
